# Role of thoracic radiotherapy for extensive-stage small cell lung cancer in the era of immunotherapy: a review of current evidence

**DOI:** 10.3389/fimmu.2026.1769165

**Published:** 2026-04-29

**Authors:** Zhipeng Li, Xiao Lei, Xingdong Guo, Qiduo He, Yanan Han, Pei Zhang, Lehui Du, Baolin Qu

**Affiliations:** 1 Department of Radiation Oncology, The First Medical Center of Chinese PLA General Hospital, Beijing, China; 2 Medical School of Chinese PLA, Beijing, China; 3 Department of Radiation Oncology, The Ninth Medical Center of Chinese PLA General Hospital, Beijing, China

**Keywords:** biomarkers, chemotherapy, extensive-stage small cell lung cancer, immunotherapy, thoracic radiotherapy

## Abstract

Extensive-stage small cell lung cancer (ES-SCLC) is an aggressive malignancy with an extremely poor prognosis. For a long time, platinum-based chemotherapy combined with etoposide has been the primary treatment option. Although the initial response rate is high, the vast majority of patients face the dilemma of rapid recurrence and drug resistance. In recent years, the application of immunotherapy has brought about a significant breakthrough in the treatment of ES-SCLC. Multiple Phase III clinical trials have demonstrated that combining immune checkpoint inhibitors with traditional chemotherapy regimens as first-line treatment significantly improves the median overall survival (OS) and progression-free survival (PFS) in patients, while maintaining manageable safety profiles. Therefore, chemotherapy combined with immunotherapy has become the new standard for first-line treatment of ES-SCLC worldwide. However, the absolute survival benefit from immunotherapy remains limited. Against this backdrop, thoracic radiotherapy (TRT), as an effective local treatment modality, shows potential for further survival gains. The combination of chemoimmunotherapy and TRT is emerging as a key area of current clinical exploration. However, the characteristics of the patient population that may benefit most from this treatment modality, as well as the optimal dose and timing of TRT, remain under investigation. Furthermore, the predictive value of previously discussed biomarkers in this combination therapy strategy for ES-SCLC remains unclear. Therefore, this paper reviewed recent advances in treatment strategies and candidate biomarkers for ES-SCLC, with a particular focus on the evolving role of thoracic radiotherapy in the era of immunotherapy.

## 1 Introduction

Lung cancer is currently the leading cause of cancer incidence and mortality worldwide, posing a severe threat to human health and survival (1). Small cell lung cancer (SCLC) is a highly malignant neuroendocrine tumor, accounting for approximately 13% to 15% of all lung cancers. The Veterans Administration Lung Study Group (VAISG) classifies SCLC into limited-stage small cell lung cancer (LS-SCLC) and extensive-stage small cell lung cancer (ES-SCLC) based on whether all lesions can be safely encompassed within a single radiation field (2). However, SCLC exhibits biological characteristics such as high invasiveness, rapid growth, and a tendency to metastasize to distant sites at an early stage. Consequently, approximately 70% of patients are diagnosed at an extensive stage at initial presentation, with a 2-year survival rate below 5% (3, 4). The treatment of ES-SCLC remains one of the most challenging problems in clinical oncology to this day.

The vast majority of patients with ES-SCLC have distant organ metastases, which fundamentally dictates a treatment strategy centered on systemic therapy. Over the past 30 years, treatment research for ES-SCLC has progressed slowly, with etoposide combined with platinum-based chemotherapy remaining the standard first-line treatment regimen (5). Although patients respond sensitively to initial treatment, with an objective response rate reaching approximately 70%, most patients experience intrathoracic recurrence or progression within one year (6, 7). The landmark positive results from the CASPIAN and IMpower133 studies have transformed this therapeutic landscape. Findings from both trials demonstrate that adding immunotherapy to first-line chemotherapy delivers superior outcomes in terms of survival benefit, tumor response, and long-term survival prognosis (8, 9). Mechanistically, tumor cells can evade the immune system through multiple pathways, including inducing immune suppression, modulating the tumor microenvironment, and binding immune checkpoints. This enables them to avoid detection and elimination by the immune system, allowing them to proliferate within the body (10). However, immune checkpoint inhibitors (ICIs) can reverse this phenomenon. ICIs block specific molecular signals, thereby exposing tumor cells and enabling the immune system to recognize and eliminate them. From then on, the treatment strategy of chemotherapy combined with immunotherapy gradually replaced chemotherapy alone, officially ushering in the era of immunotherapy for ES-SCLC.

Unsatisfactorily, even the most promising treatment regimens combining first-line chemotherapy with programmed cell death ligand 1 (PD-L1) or programmed cell death protein 1 (PD-1) inhibitors yield only limited efficacy in ES-SCLC. The median overall survival (OS) for ES-SCLC patients receiving chemoimmunotherapy is extended by only about 3 months compared to chemotherapy alone (11, 12). Thoracic radiotherapy (TRT), as a key treatment modality for lung cancer, has been proven since the era of chemotherapy to confer survival benefits for ES-SCLC patients (6, 13, 14). The CREST study demonstrated that among ES-SCLC patients who responded well to platinum-based chemotherapy, consolidation TRT significantly increased the 2-year OS rate by 10% (13% vs. 3%, P = 0.004) and reduced the rate of intrathoracic recurrence (79.8% vs. 43.7%, P<0.0001) (6). This study established the role of thoracic radiotherapy in ES-SCLC. However, it has not been incorporated into the treatment regimens of various studies in the era of immunotherapy. In recent years, as the synergistic effects of radiotherapy and immunotherapy have gradually been elucidated, the question of whether adding TRT to first-line chemoimmunotherapy could further improve survival outcomes for patients with ES-SCLC has attracted the attention of researchers (15). Therefore, we reviewed the current application status and research progress of TRT in ES-SCLC during the era of immunotherapy, and explored potential biomarkers for the efficacy of this combined treatment. We believe that classifying ES-SCLC patients based on biomarkers holds significant importance for implementing personalized lung cancer treatment.

## 2 The theoretical basis of combining radiotherapy with immunotherapy

**Figure 1:**
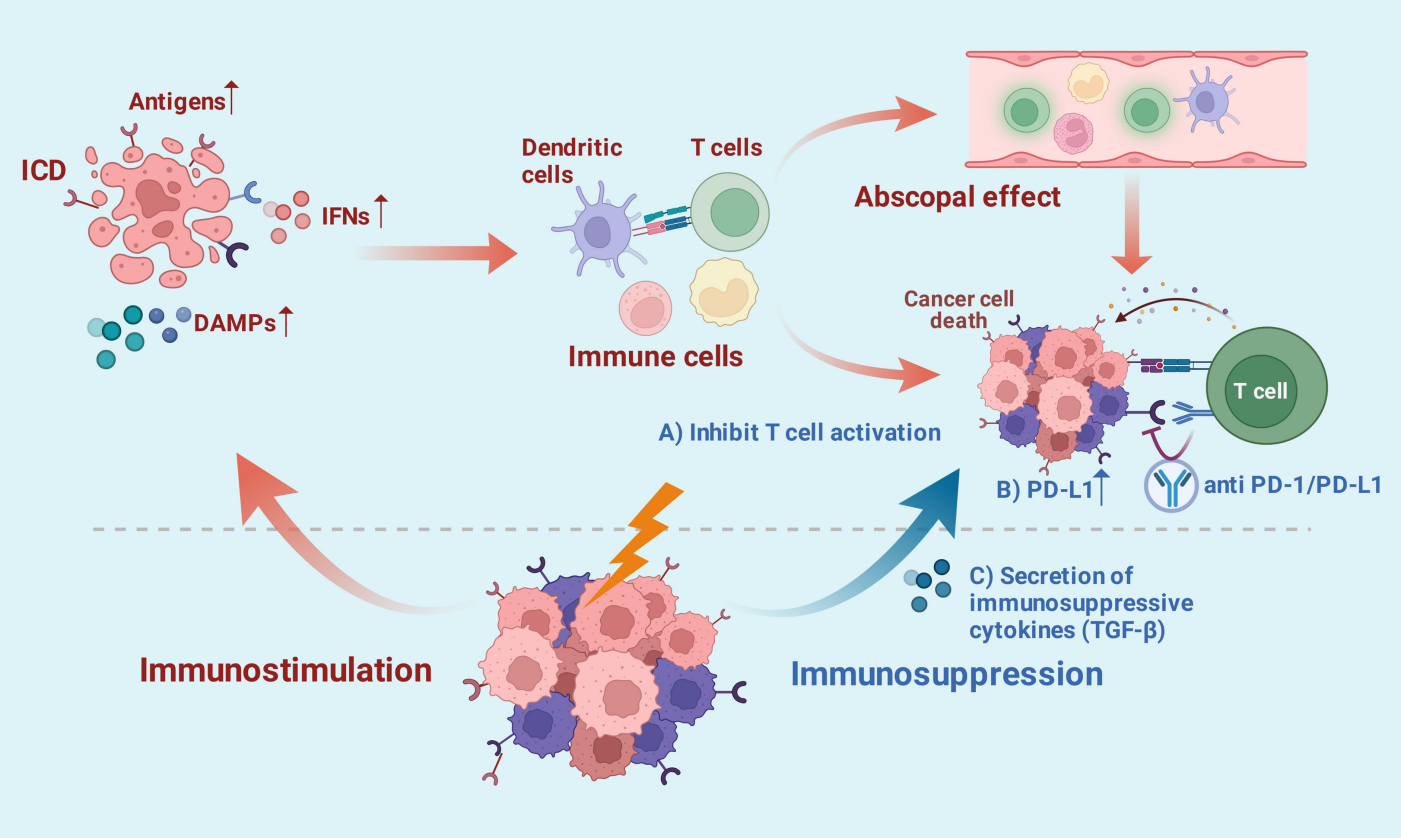
Radiotherapy generates bidirectional immune effects in tumor treatment. Created in BioRender. Li, Z. (2026) https://BioRender.com/slrtkft.

Multiple preclinical studies have revealed that the interaction between radiotherapy and immunotherapy can produce synergistic antitumor effects, with the underlying mechanisms being complex and intricate. Radiation therapy can induce bidirectional immune responses, either stimulating or suppressing the immune system (**Figure 1**). On one hand, radiotherapy can induce tumor cell damage and immunogenic cell death (ICD), leading to the release of damage-associated molecular patterns (DAMPs) and the activation of pattern recognition receptors (PRRs) (16). Further studies indicate that radiation can also remodel the tumor immune microenvironment by increasing tumor antigen exposure, promoting the release of proinflammatory factors, and upregulating the expression of major histocompatibility complex (MHC) class I molecules and other adhesion molecules (17). Ultimately, it can stimulate immune responses and promote the killing of tumor cells by immune cells such as dendritic cells, natural killer (NK) cells, macrophages, and T cells (16). Additionally, combining radiotherapy with immunotherapy in patients with advanced tumors can stimulate systemic antitumor immunity and lead to the shrinkage of non-irradiated lesions distant from the tumor treatment site, which is known as the abscopal effect (18). On the other hand, radiotherapy may also exert immune-suppressive effects through mechanisms such as suppressing CD8+ T cell function, promoting the release of immune-suppressive inflammatory cytokines, upregulating the expression of PD-L1, and inhibiting T cell activation (19). However, ICIs can synergistically enhance the antitumor effects of radiotherapy by promoting local and systemic immune responses, thereby partially counteracting the immune-suppressive effects induced by radiotherapy.

It is noteworthy that the fractionation regimen of radiotherapy is closely associated with its immunomodulatory effects. Research indicates that single-dose radiotherapy below 2 Gy can mobilize innate and adaptive immune responses, promoting the activation and infiltration of immune effector cells such as CD4+ and CD8+ T cells (20). Differently, single high-dose (5–10 Gy) radiotherapy and hypo-fractionated radiotherapy can directly induce tumor cell death through biological processes such as mitotic death, apoptosis, autophagy, and senescence. On the other hand, antitumor immunity can be stimulated by enhancing tumor immunogenicity, promoting immune cell infiltration, inducing immunogenic cell death, and increasing the ratio of effector T cells to regulatory T cells (Tregs) (21). However, other studies have suggested that irradiation exceeding the threshold dose for DNA three prime repair exonuclease 1 (TREX1) may lead to insufficient recruitment and activation of dendritic cells (DCs) and a significant reduction in CD8+ T cell numbers. This ultimately results in a decreased incidence of abscopal effects in combination therapy (22). In summary, the synergistic effects of radiotherapy and immunotherapy offer new possibilities for overcoming tumor immune tolerance and treating advanced tumors. However, the efficacy of this combination therapy may be closely related to factors such as the fractionation regimen of radiotherapy, the volume of irradiation field, and the timing of immunotherapy administration.

## 3 Clinical advances in radiotherapy combined with immunotherapy for lung cancer

In recent years, immunotherapy has significantly transformed the treatment landscape for lung cancer. In the treatment of locally advanced non-small cell lung cancer (LA-NSCLC), sequential immunotherapy following concurrent chemoradiotherapy (CRT) has become the standard treatment regimen for most patients. The PACIFIC study first reported that among LA-NSCLC patients who remained progression-free after CRT, those receiving durvalumab consolidation therapy demonstrated longer PFS compared to those receiving placebo (16.8 vs. 10.6 months), indicating that the addition of immunotherapy confers a significant survival benefit (23). Additionally, multiple clinical studies have also explored the therapeutic efficacy of chemotherapy-free radioimmunotherapy regimens in LA-NSCLC, with results also demonstrating favorable efficacy and safety profiles (24–26). Similarly, in patients with advanced or metastatic NSCLC following biomarker screening, the combination of radiotherapy and immunotherapy has also been demonstrated to potentially be more effective than chemotherapy (27, 28). In conclusion, the combination of radiotherapy and immunotherapy shows tremendous potential in the treatment of NSCLC.

However, for small cell lung cancer, the evidence supporting the efficacy of radiotherapy combined with immunotherapy is limited. The most fundamental issue may stem from the highly immune-suppressive microenvironment of the SCLC. Most SCLC tumors lack cytotoxic T cell infiltration and instead harbor immune-suppressive cells such as M2-type tumor-associated macrophages and regulatory T cells, forming what is termed an immune desert (29). Additionally, the expression downregulation and function loss of MHC-I in SCLC, along with the low PD-L1 expression, may also contribute to the limited efficacy of immunotherapy (30). Given the biological characteristics of SCLC, high tumor burden and rapid progression may overwhelmingly deplete the immune system, leading to functional T-cell exhaustion and hindering the establishment of effective antitumor immunity. The high tumor heterogeneity of SCLC may also impact immune system function. Recent studies have classified SCLC into distinct molecular subtypes (including SCLC-A, N, P, and I), which differ in cellular origin, gene expression profiles, and response to treatment. Under the pressure of immunotherapy, tumor cells may undergo subtype conversion, thereby altering their antigen expression and interactions with the microenvironment, leading to treatment resistance.

Despite this, the ADRIATIC trial has for the first time demonstrated significant benefits of immune consolidation therapy in LS-SCLC, bringing new hope for the application of immunotherapy combined with TRT in small cell lung cancer (31). In this landmark study, consolidation therapy with durvalumab after concurrent chemoradiotherapy (cCRT) significantly improved OS (55.9 vs. 33.4 months; HR = 0.73, P = 0.01) and PFS (16.6 vs. 9.2 months; HR = 0.76, P = 0.02) in patients with LS-SCLC. This finding was largely consistent with the PACIFIC trial (23). It suggested that, regardless of histologic type, chemoradiotherapy might prime an antitumor immune response through immunogenic cell death, neoantigen release, and upregulation of MHC-I molecules, thereby altering the tumor microenvironment and increasing sensitivity to subsequent immunotherapy. However, extrapolating this paradigm to ES-SCLC requires attention to key differences between limited-stage and extensive-stage disease. First, the target volume and radiation field differ. In LS-SCLC, cCRT typically covers the primary tumor and all visible lesions. In contrast, consolidative TRT is usually directed only at residual intrathoracic disease following systemic therapy in ES-SCLC, while extrathoracic metastases are not irradiated (4). This implies that the abscopal effect or *in situ* vaccine effect of TRT may be confined to thoracic lesions, whereas unirradiated distant metastases may contribute to immune resistance (32). Second, the tumor microenvironment may differ. Compared with ES-SCLC, LS-SCLC generally has a lower tumor mutational burden and less systemic immunosuppression caused by extrathoracic lesions. Patients with ES-SCLC often have liver, bone, or brain metastases, which can create a more profoundly immunosuppressive environment, thereby potentially attenuating the immune priming effect induced by radiotherapy (33). Third, the current treatment standard for ES-SCLC combines ICIs with induction chemotherapy as first-line therapy, with or without the addition of TRT during the consolidation phase (8). Consequently, whether TRT should be administered before ICIs to prime the immune response remains unclear.

## 4 Treatment strategies of TRT plus first-line chemoimmunotherapy for ES-SCLC

### 4.1 Treatment efficacy and characteristics of beneficiary populations

Immunotherapy has gradually become the first-line systemic treatment for advanced SCLC. Whether the addition of TRT can reverse the immune desert state in SCLC has become a hot research topic. Multiple retrospective studies have evaluated the value of TRT as a consolidation treatment for first-line chemoimmunotherapy in ES-SCLC. But the substantial patient heterogeneity inherent in retrospective analyses is worthy of attention (**Table 1**).

**Table 1:** Published clinical studies regarding first-line chemoimmunotherapy combined with TRT in patients with ES-SCLC.

Author (year)	Median age (years)	Gender (Male %)	ECOG (0-1%)	Brain metastasis (%)	Liver metastases (%)	Bone metastases (%)	TRT sequence	Dose of TRT	n (TRT/no TRT)	Types of immunotherapy	Effectiveness		Safety	
											OS (Months)	PFS (Months)	≥G3 TRAEs (%)	Pneumonitis (%)
Diamond 2022	66	35	80	10	–	–	Sequential	30-60Gy	20	Atezolizumab	16.0	6.7	0	0
J. Wu 2022	64	90.1 vs 81.8	90.9 vs 63.6	27.3 vs 45.5	–	–	Sequential	28-64Gy	22 (11/11)	Atezolizumab/durvalumab	NR vs 9.6	–	–	54.6
Z. Cai 2023 (40)	41	84.6	96.2	15.4	14.1	–	Sequential/Concurrent	–	78	–	20	9.2	29.5	23.1
M. Fang 2023	62 vs 64	92.3 vs 94.4	–	43.6	12.8 vs 43.1	20.5 vs 40.3	Sequential/Concurrent	24-60Gy	111 (39/72)	Atezolizumab/durvalumab	21 vs 12	7 vs 5	30.8 vs 22.2	–
Hoffmann 2023 (41)	63 vs 62	47.8 vs 61.1	–	30.4 vs 52.6	43.5 vs 47.4	–	Sequential	30Gy/10f	41 (23/18)	Atezolizumab/durvalumab	21.1 vs 10.8	–	–	–
Kim 2023 (35)	66	90.2	83	32	–	–	Sequential	52-66Gy	41 (6/35)	Atezolizumab	NR vs 12.6	5.3 vs 4.9	–	50.0
L. Li 2023 (34)	63	86.1	94	19	22	11	Sequential	37.5-60Gy	36	Atezolizumab/durvalumab	NR	12.8	5.56	8.3
Y. Li 2023 (34)	59 vs 63	78.7 vs 88.7	–	36.2 vs 32.1	21.3 vs 37.7	25.5 vs 24.5	Sequential	30-60Gy	100 (47/53)	Atezolizumab/durvalumab	21.8 vs 24.3	9.1 vs 8.8	–	76.6
C. Liu 2023 (46)	58	97.5	100	7.5	15	–	Concurrent	30-45Gy	40	Atezolizumab	NR	8.6	22.50	12.5
Longo 2023 (51)	64 vs 71	55.9 vs 60.7	–	17 vs 15	19 vs 20	31 vs 29	Sequential	20-60Gy	120 (59/61)	Atezolizumab/durvalumab	–	–	1.7 vs 3.3	6.8 vs 3.3
J. Peng 2023 (36)	63 vs 61	75.4 vs 77.2	91.2 vs 96.5	24.6 vs 29.8	24.6 vs 29.8	–	Sequential/Concurrent	30-60Gy	114 (57/57)	Atezolizumab/durvalumab	24.1 vs 18.5	9.5 vs 7.2	–	35.1 vs 15.8
Z. Xie 2023 (60)	62 vs 63	77.8 vs 83.6	80 vs 79.5	31.1 vs 27.3	28.8 vs 28.8	13.3 vs 39.7	Sequential	30-60Gy	118 (45/73)	–	22.7 vs 14.7	9 vs 5.9	37.8 vs 32.9	4.4 vs 1.4
X. Mu 2024 (43)	64	83.6 vs 86.4	98.2 vs 100	12.7 vs 20.5	25.5 vs 40.9	–	Sequential	14-66Gy	99 (55/44)	–	28 vs 16	13 vs 5	78.2 vs 40.9	–
D. Chen 2024 (45)	63	83.6	100	33	31	–	Sequential	≥30Gy/10f or ≥50Gy/25f	67 (45/22)	Adebrelimab	22.9 vs 13.4	11.3 vs 4.1	62.2 vs 50.0	35.6 vs 4.6
L. Chen 2024 (45)	–	–	–	–	–	–	Sequential	45Gy/15f or 60Gy/30f	110 (38/72)	Durvalumab	17.2 vs 12.3	9.1 vs 5.9	–	–
Y. Yao 2024 (38)	62	78.8 vs 82.7	74.7 vs 79.6	33.3 vs 35.7	24.2 vs 23.5	24.2 vs 27.6	Sequential	30-66Gy	197 (99/98)	–	21.67 vs 16.60	10.76 vs 7.63	4.0 vs 4.1	16.2 vs 9.2
L. Zheng 2024 (44)	65.25	98.3 vs 84.8	82.8 vs 71.2	36.2	–	–	Sequential	30-60Gy	124 (58/66)	Tislelizumab/serpluliumab/durvalumab/adebrelimumab	15.5 vs 10.5	–	–	–
Y. Zhang 2024	58	96.7	100	10	20	–	Concurrent	15Gy/5f	344 (168/176)	–	20.6 vs 13.4	–	–	–
Kim 2025 (56)	66	81.8	100	–	–	–	Sequential	52.5Gy/25f	22	Atezolizumab	26	–	–	22.7
Monaca 2025 (39)	64 vs 66	48.6 vs 56.4	96.4 vs 93.8	17.1 vs 19.1	25.2 vs 42.2	–	Sequential	20-60Gy	336 (111/225)	Atezolizumab/durvalumab	13.5 vs 11	8.1 vs 6.1	–	–
L. Zhou 2025 (49)	58.5	87.5	100	5.4	17.9	44.6	Concurrent	15Gy/5f	56	Atezolizumab	16.9	6.9	91.1	–
Cakš 2025	66 vs 66	56.6 vs 55.6	80.4 vs 72.8	19.6 vs 19.8	13 vs 52.5	21.7 vs 37.7	Sequential	20-60Gy	208 (46/162)	Atezolizumab/durvalumab	17.0 vs 10.8	9.7 vs 6.1	26.1 vs 21.3	6.5 vs 0
Y. Gao 2025	60 vs 62	66.7 vs 81.25	91.7 vs 93.7	10 vs 22.3	33.3 vs 23.2	20 vs 45.5	Sequential/Concurrent	15-63Gy	172 (60/112)	–	24.2 vs 15.9	11.3 vs 6.6	–	–
N. Yao 2025	63 vs 65	69.0 vs 79.1	93.1 vs 83.7	–	–	–	Sequential	30-60Gy	72 (29/43)	Tislelizumab/serpluliumab/durvalumab/adebrelimumab	26.7 vs 16.3	11.5 vs 8.0	58.6 vs 58.1	13.8 vs 7.0
C. Zhang 2025 (49)	62.5	81.25 vs 92.3	97.9 vs 88.5	8.3 vs 15.4	12.5 vs 32.7	14.6 vs 36.5	Sequential	30-60.2Gy	100 (48/52)	Atezolizumab/durvalumab	26.0 vs 17.0	10.0 vs 6.0	–	20.8 vs 2.0
Q. Zhang 2025 (49)	63 vs 63	70.4 vs 82.1	87.7 vs 77.2	34.6 vs 32.5	24.7 vs 30.1	27.2 vs 24.4	Sequential	30-60Gy	204 (81/123)	–	NA vs 20.8	10.9 vs 8.0	60.5 vs 82.9	25.9 vs 37.4

First, patient selection criteria. Most of the studies mainly included male patients with an ECOG score of 0–1 and a median age of over 60 years old. The study of Li et al. included 36 patients with ES-SCLC who received TRT following 4–6 cycles of chemoimmunotherapy. And all the patients maintained progression-free disease before radiotherapy. The 1-year OS rate was 80.2%, and the 2-year OS rate was 53.3% (34). This preliminary finding suggested that the addition of TRT conferred a potential survival benefit in ES-SCLC patients who achieved a favorable response to first-line chemoimmunotherapy. Additionally, a retrospective study from South Korea demonstrated that high-dose thoracic consolidation radiotherapy effectively reduced intrathoracic tumor recurrence in certain patients while maintaining acceptable treatment-related toxicity. In this study, 22 patients with ES-SCLC demonstrated favorable tumor response following first-line chemoimmunotherapy, but those with oligoprogressive disease confined to the thoracic cavity were also included. Results showed that only one patient experienced local recurrence within the radiation field following TRT. Most importantly, 10 patients with oligoprogression in the thoracic cavity remained stable without further intrathoracic recurrence (35). This clinical experience suggests that the addition of TRT may improve long-term survival rates in carefully selected ES-SCLC patients.

Subsequently, several studies have been conducted to investigate the impact of baseline metastatic burden. A retrospective study enrolled 114 patients with ES-SCLC who received first-line chemoimmunotherapy with or without TRT. Results demonstrated significantly prolonged OS in patients receiving TRT (24.1 vs. 18.5 months, P = 0.016) (36). Importantly, over 80% of patients receiving TRT had fewer than three metastatic lesions, suggesting that patients with lower extra-thoracic tumor burden are more likely to benefit from combined TRT and chemoimmunotherapy. Furthermore, another retrospective study failed to demonstrate an association between the addition of TRT and survival benefit, likely due to the significantly higher rate of extra-thoracic progression in the TRT group (91.4% vs. 67.6%) (37). A subsequent multicenter retrospective study also confirmed that the addition of TRT significantly improved the median PFS (10.76 vs. 7.63 months, P = 0.014) and OS (21.67 vs. 16.6 months, P = 0.009). Subgroup analysis further clarified that the addition of TRT conferred survival benefits for those harboring fewer than two metastatic sites, whereas it proved ineffective for those with more than two metastatic sites. Moreover, patients with liver metastases did not derive survival benefit from TRT, whereas those with bone or brain metastases experienced PFS improvement (38). Recently, a multicenter retrospective study from Italy proposed that baseline liver metastasis and stable disease (SD) following chemoimmunotherapy were independent predictors of shorter PFS in patients with ES-SCLC. Subgroup analysis further revealed that among patients receiving TRT, those without baseline liver metastasis demonstrated longer tumor-free survival (39). Therefore, identifying the specific patients who can benefit from TRT in the era of immunotherapy is both clinically significant and highly challenging.

### 4.2 The optimal dose and intervention timing of TRT

A number of previous retrospective studies explored the optimal timing and fractionation regimen for TRT. Regarding the timing of intervention, patients received sequential TRT after 4–6 cycles of first-line chemoimmunotherapy in most studies, whereas several studies administered concurrent TRT during the chemoimmunotherapy. And Cai et al. found no significant difference between early TRT (≤3 cycles of chemoimmunotherapy) and late TRT (>3 cycles) in PFS and OS of ES-SCLC patients (40). However, several studies demonstrated that patients receiving consolidative TRT exhibited better survival outcomes than those who receive TRT in case of progression (41, 42). Studies also investigated the impact of fractionation regimen on the prognosis of ES-SCLC patients. The study by Mu et al. indicated that low-dose radiotherapy below 30 Gy or high-dose radiotherapy exceeding 30 Gy did not affect the OS and PFS in ES-SCLC patients (43). Similarly, Zheng et al. demonstrated that in patients with ES-SCLC receiving first-line chemoimmunotherapy combined with TRT, the survival outcomes were unaffected by whether the BED was greater than or less than 60 Gy (44). Interestingly, a retrospective study also found no significant differences in objective response rates or survival outcomes between the conventional fractionated, hyperfractionated, and hypofractionated groups (44).

Notably, several prospective studies conducted in recent years also explored various fractionation regimens for TRT. In a phase II trial, ES-SCLC patients receiving 4–6 cycles of adebrelimab combined with etoposide plus carboplatin (EC)/cisplatin (EP) underwent sequential TRT (≥30 Gy in 10 fractions or ≥50 Gy in 25 fractions). Moreover, TRT should ideally commence within 6 weeks after chemotherapy and no later than 7 weeks post-chemotherapy. The results demonstrated significant survival benefits and acceptable safety (45). Additionally, two other prospective studies explored different treatment modalities, investigating the efficacy and safety of concurrent TRT during first-line chemoimmunotherapy. In a multicenter phase II study, ES-SCLC patients received two cycles of atezolizumab combined with EC/EP chemotherapy. Subsequently, progression-free patients concurrently received hypofractionated TRT (30–45 Gy, 3 Gy/fraction) alongside chemoimmunotherapy, followed by atezolizumab maintenance therapy. Results demonstrated a median PFS of 8.6 months, with only 7 Grade 3 adverse events and 2 Grade 4 adverse events occurring among 40 patients (46). Besides, another study also demonstrated favorable efficacy when low-dose radiation therapy (LDRT; 15 Gy in 5 fractions) was administered concurrently during the induction phase of first-line chemoimmunotherapy for ES-SCLC. The objective response rate (ORR) was 87.5% and the disease control rate (DCR) was 94.6% (47). The study offers a new therapeutic option and direction for the treatment of ES-SCLC.

### 4.3 Safety assessment

It appears certain that first-line chemoimmunotherapy combined with TRT yields survival benefits for ES-SCLC patients, while its safety profile remains another key concern. The study by Mu et al. indicated that the rate of Grade 3–4 adverse reactions in ES-SCLC patients receiving TRT after first-line chemoimmunotherapy did not significantly differ from those not receiving TRT (43). Another study similarly demonstrated that consolidation TRT following four cycles of chemoimmunotherapy did not increase adverse reactions of any grade (P = 0.874). Furthermore, the study indicated that the most common adverse reactions in the TRT group were radiation esophagitis, gastrointestinal reactions, and hematologic reactions, all of which were tolerable (38). Notably, the TREASURE study was a prospective clinical trial designed to evaluate the efficacy of TRT combined with maintenance immunotherapy in ES-SCLC patients following standard first-line chemoimmunotherapy. However, it was terminated early due to unexpected increased toxicity (48). It suggested that whether the addition of TRT is truly safe warrants our utmost attention and further investigation.

It was noteworthy that in past immunotherapy trials, the addition of thoracic radiotherapy was not recommended. This was because the combination of radiotherapy and immunotherapy may increase the incidence of pneumonia, thereby causing greater harm to patient survival (49, 50). However, results from several retrospective studies indicated that the addition of consolidation TRT did not increase the incidence of pneumonia in ES-SCLC patients following the first-line chemoimmunotherapy, preliminarily establishing the safety profile of TRT with respect to pneumonia (38, 51). Interestingly, some studies reported opposite results. Peng et al. reported that adding TRT to chemotherapy combined with immunotherapy significantly improved PFS and OS, but also significantly increased the incidence of treatment-related pneumonia (P = 0.018) (36). Among the 57 patients who underwent TRT, 15 received concurrent TRT during the first two cycles of chemoimmunotherapy, which may be associated with the high incidence of pneumonia. Subsequently, Chen et al. also reported a higher incidence of pneumonia (16/45) in the group receiving TRT after chemotherapy combined with adebrelimab compared to the group without TRT (1/22), although the study did not report the statistical significance value (45). Furthermore, a recent study clearly demonstrated that compared with the chemoimmunotherapy alone group, patients in the TRT group experienced similar rates of Grade 3 or higher adverse events (26.1% vs. 21.3%), but a significantly higher incidence of pneumonia (6.5% vs. 0%, p = 0.001) (52). Therefore, we suppose that TRT may not increase the occurrence of adverse reactions on a whole, but it may increase the toxic effects on the lungs. In Addition, Yao et al. found that increased radiation doses of TRT correlate with higher rates of pneumonia (38). This suggests that when determining the optimal radiation dose for TRT, we should pay attention to the impact on pneumonia.

## 5 Candidate biomarker in the era of chemoimmunotherapy for ES-SCLC

As treatment strategies for small cell lung cancer continue to evolve, it has become crucial to better identify the patient subgroups that benefit from different therapeutic approaches. Tissue and blood biomarkers have become essential tools for guiding treatment decisions in patients with advanced lung cancer. By detecting these biomarkers, patients can be categorized into distinct subgroups, providing crucial evidence for selecting the optimal therapeutic approach. Biomarkers developed during the era of traditional chemotherapy are not suitable for guiding immunotherapy decisions in SCLC. Therefore, exploring new predictive biomarkers in the era of chemoimmunotherapy for ES-SCLC holds significant clinical value.

### 5.1 PD-L1 expression

Compared to NSCLC cells, PD-L1 expression is lower in SCLC cells, with the reported expression rate ranging from 10% to 40% (53, 54). Previous studies suggest that PD-L1 expression does not appear to be a reliable predictor of treatment response in ES-SCLC. The IMpower133 study evaluated PD-L1 expression in 34% of the intention-to-treat population, revealing survival benefits with atezolizumab plus chemotherapy in both the PD-L1 ≥ 1% and PD-L1 < 1% subgroups (8). Consistent with the IMpower133, the CASPIAN study also demonstrated that durvalumab combined with platinum-based chemotherapy yielded similar overall survival benefits regardless of PD-L1 expression status (55). Additionally, the KEYNOTE-604 study analyzed OS and PFS across all PD-L1 subgroups. Results demonstrated that in PD-L1 subgroups, pembrolizumab plus etoposide showed comparable benefits to etoposide monotherapy (11). In summary, PD-L1 expression has not been established as a predictive factor for benefit from first-line chemoimmunotherapy in ES-SCLC. However, both preclinical and clinical studies have demonstrated that radiotherapy is an effective inducer of PD-L1 expression, capable of upregulating PD-L1 expression in tumor cells through multiple biological signaling pathways (56). Therefore, it is worth our attention whether the addition of TRT in the first-line chemoimmunotherapy regimen for ES-SCLC can improve the predictive ability of PD-L1 expression in survival.

### 5.2 Tumor mutational burden

In recent years, with the widespread adoption of precision medicine concepts and the discovery of molecular biomarkers, treatment strategies for lung cancer have shifted from traditional standardized approaches to personalized therapies based on individual genetic characteristics. Among these, tumor mutational burden (TMB) refers to the number of somatic mutations within a tumor, which can indirectly reflect the tumor’s capacity to generate neoantigens (57). TMB correlates with the efficacy of immunotherapy across multiple tumor types, including lung cancer (58). However, the use of TMB to predict immunotherapy response in SCLC remains controversial, with inconsistent results across different studies. In the IMpower133 study, 92.8% of patients had available blood-based TMB (bTMB). Results showed that patients receiving first-line chemotherapy plus atezolizumab experienced similar survival improvements regardless of bTMB status (59). Subsequently, in the CASPIAN study, 35% of patients obtained tumor tissue TMB (tTMB). However, the 10mut/Mb threshold proposed in the study also failed to predict survival benefit in patients receiving first-line chemoimmunotherapy (60). Notably, a prospective, phase 2 study by Chen et al. reported different results. This study demonstrated that high tTMB levels (≥10 Mut/Mb) correlated with improved prognosis in patients with ES-SCLC receiving first-line chemoimmunotherapy followed by sequential radiotherapy. Furthermore, patients with high tTMB also exhibited a numerical survival benefit (P = 0.074) when treated with first-line chemotherapy combined with immunotherapy (45). However, another recent Phase II prospective study on first-line chemoimmunotherapy combined with concurrent TRT reported negative results. The study concluded that there were no correlations in PFS and OS assessed by TMB status in tumor tissue or peripheral blood samples (high vs low, cutoff by median) (47).

### 5.3 Genetic alterations

Recent studies have described the heterogeneity of SCLC to identify patients most likely to benefit from immunotherapy (61). Gay et al. classified SCLC into four distinct subtypes based on the relative expression levels of three transcription factors using non-negative matrix factorization (NMF) (62). They were SCLC-A with ASCL1 as the dominant factor, SCLC-N with NEUROD1 as the dominant factor, SCLC-P with POU2F3 as the dominant factor and SCLC-I with low expression of all three transcription factors. Among these, the SCLC-I subtype was characterized by high expression of inflammatory genes and infiltration of immune cells, suggesting it may possess potential advantages for immunotherapy. Additionally, results from the IMpower133 study demonstrated that patients with SCLC-I subtype treated with atezolizumab/etoposide had a prolonged OS compared with those receiving etoposide alone (median OS: 18.2 months vs. 10.4 months) and the survival benefit was significantly better than that of other subtypes (63). This finding suggested that the SCLC-I subtype might serve as a potential predictive biomarker for chemotherapy combined with immunotherapy of SCLC.

Furthermore, previous studies suggested that dual mutations in TP53/RB1 are major drivers of SCLC (64). And co-mutations in TP53/RB1 induced an immune-suppressive microenvironment, potentially hindering response to immune checkpoint inhibitors in SCLC (65). Recently, Chen et al. similarly confirmed this conclusion in ES-SCLC patients receiving first-line chemoimmunotherapy with or without sequential radiotherapy. Research findings indicated that the co-occurrence of TP53/RB1 mutations in tumor tissues correlated with unfavorable overall survival in all patients receiving first-line chemoimmunotherapy. Notably, the study also monitored the dynamic changes in circulating tumor DNA (ctDNA) during treatment. Consistent with the findings from genomic profiling within the organization, the detection of TP53/RB1 double mutations in baseline ctDNA was associated with poorer prognosis in all patients receiving first-line chemoimmunotherapy and those receiving first-line chemoimmunotherapy combined with thoracic radiotherapy. Additionally, ctDNA levels prior to thoracic radiotherapy, which reflected residual cancer cells following chemoimmunotherapy treatment, also was proposed to correlate with PFS in ES-SCLC patients. Accordingly, patients with cleared ctDNA or absence of TP53/RB1 double mutations were more likely to benefit from first-line immunotherapy followed by radiotherapy (45). Subsequently, the study by zhou et al. also collected tumor tissue samples and peripheral blood samples from ES-SCLC patients receiving first-line chemoimmunotherapy with concurrent TRT and performed gene sequencing (47). The results indicated that the most frequently mutated genes were TP53 and RB1, which were the most common tumor suppressor gene mutations in SCLC (66). However, according to CASPIAN trial, the mutational status of TP53, RB1, and other genes that were altered in ≥ 5% of patients was not linked to the treatment response to first-line chemoimmunotherapy in ES-SCLC patients (60).

### 5.4 Systemic inflammatory markers

With the advent of the immunotherapy era, multiple studies have further revealed a close association between inflammatory hematologic markers and the efficacy of immunotherapy in SCLC (67, 68). However, there is currently a lack of specific exploration across different tumor stages and treatment modalities. Previous studies have demonstrated that novel inflammatory markers such as the lymphocyte-to-C-reactive protein ratio (LCR), systemic inflammatory response index (SIRI), and hemoglobin-to-red cell distribution width ratio (HRR) serve as independent prognostic factors for survival in ES-SCLC patients (69). A study of second-line or later immunotherapy in ES-SCLC patients demonstrated that patients with a neutrophil-to-lymphocyte ratio (NLR) <5 at 6 weeks post-treatment exhibited longer median PFS compared to those with an NLR ≥5 (HR = 0.29, P = 0.04). This suggests that post-treatment NLR levels may serve as a potential predictive indicator of immunotherapy efficacy in ES-SCLC patients (70). However, a study suggest that chemotherapy may alter the immune microenvironment, leading to different responses to immunotherapy among patients (71). Therefore, predictive markers for immunotherapy in ES-SCLC during second-line or subsequent-line treatment may not be applicable for predicting response in patients receiving first-line chemotherapy combined with immunotherapy. Exploring biomarkers under specific treatment regimens may enable more precise prediction of survival in patients with ES-SCLC.

A study involving 17 patients with ES-SCLC receiving first-line treatment with tislelizumab plus chemotherapy found that patients who responded positively to treatment exhibited higher levels of inflammatory markers compared to non-responders (72). Results from a Phase II study indicated that pre-treatment PLR was an independent prognostic factor for survival in patients with ES-SCLC receiving first-line chemoimmunotherapy. Patients with ES-SCLC and low PLR (≤119.23) demonstrated superior prognosis compared to those with high PLR (1-year OS: 87% vs. 42%, p = 0.0004) (73). Another retrospective study found that a decreased C-reactive protein to lymphocyte ratio (CLR) in SCLC patients receiving chemoimmunotherapy was associated with prolonged PFS and improved objective response rates, suggesting that dynamically monitored CLR may serve as a potential prognostic biomarker (74). Moreover, Pan-Immune-Inflammation Value (PIV) is calculated using the formula: (platelet count × neutrophil count × monocyte count)/lymphocyte count. The PILE score is a prognostic scoring system comprising three parameters: PIV, lactate dehydrogenase (LDH), and Eastern Cooperative Oncology Group Performance Status. A study indicated that among ES-SCLC patients receiving chemoimmunotherapy, those with elevated PIV levels (PIV ≥ 581.95) exhibited shorter PFS and OS, while patients with high PILE score demonstrated poorer treatment efficacy and survival outcomes (75). Subsequently, a new study further proposed that the combination of the systemic immune inflammation index (SII) with PD-L1 (SP142) expression was more effective in predicting survival and disease progression in ES-SCLC patients receiving chemoimmunotherapy (76). This conclusion provided a direction for constructing survival prediction models for ES-SCLC patients undergoing first-line chemoimmunotherapy. Interestingly, a recent prospective study preliminarily demonstrated that ES-SCLC patients with progressive disease (PD) after first-line immunotherapy combined with concurrent TRT exhibited fewer lymphocytes and higher NLR values (47). However, current exploration of inflammatory biomarkers in TRT combination therapy strategies remained scarce, representing a critical area for future research.

### 5.5 Dynamic changes of immune cells and the effective dose of immune cells

Research has reported that the moderate response to immunotherapy may be associated with reduced immune cell infiltration in the tumor immune microenvironment. Therefore, the degree of immune cell infiltration can predict the response to immunotherapy in patients with SCLC (77). The benefit observed in SCLC patients undergoing immunotherapy has been reported to potentially correlate with the infiltration and activation of T cells and NK cells (78). A retrospective study found that among patients with ES-SCLC receiving first-line chemoimmunotherapy, the median PFS was longer in the group with normal baseline NK cell levels compared to the group with lower baseline NK cell levels (7.0 vs. 4.6 months; P < 0.0001) (79). This finding indicates that the dynamic changes in peripheral NK cells following chemoimmunotherapy can serve as a predictive indicator of treatment efficacy. Additionally, Schmälter et al. analyzed changes in peripheral T cells, B cells, and NK cells in ES-SCLC patients receiving combination therapy with carboplatin, etoposide, and atezolizumab. The results revealed that post-treatment reduction in Th17 cells correlated with improved OS, suggesting that Th17 may also be a potential predictor (80).

It is noteworthy that radiotherapy can both release tumor antigens to enhance immune responses and potentially act as a hidden killer by inducing lymphocyte depletion. When lymphocytes are depleted during radiotherapy, ICIs become like commanders who have lost their soldiers, and the efficacy of immunotherapy is significantly limited. Therefore, we hypothesize that combining thoracic radiotherapy with the ES-SCLC chemoimmunotherapy regimen may also lead to changes in peripheral blood lymphocytes, thereby affecting the efficacy of immunotherapy and consequently influencing the survival and prognosis of ES-SCLC patients. The introduction of the concept of Effective Dose to Immune Cells (EDIC) quantifies the radiation dose received by circulating lymphocytes, providing a reference metric for radiation therapy-induced immunosuppression (81). Subsequently, Ladbury et al. refined the EDIC model and renamed it the Estimated Dose of Radiation to Immune Cells (EDRIC) (82). In previous studies, both the EDIC and EDRIC models have been widely used to quantify the systemic radiation dose to immune cells during thoracic radiotherapy. Multiple studies have confirmed that elevated EDIC/EDRIC levels correlate with poorer survival outcomes and increased distant metastasis in NSCLC, esophageal cancer, and breast cancer (83–85). However, the survival predictive role of EDIC/EDRIC in SCLC remains unclear, and there are very few related studies. A study in 2021 first proposed that EDIC could serve as an independent predictor of lymphopenia, PFS and OS in patients with LS-SCLC (86). However, the application of immunotherapy in this study was not widespread. Subsequently, the results of a recent study indicated that a higher EDIC was associated with shorter OS, PFS, and distant metastasis-free survival (DMFS) in patients with LS-SCLC, while it was unrelated to the prognosis of ES-SCLC (87). However, it is worth noting that this study included all ES-SCLC patients who received TRT without restricting patient characteristics or treatment regimens, resulting in significant patient heterogeneity. Therefore, further investigation into the role of EDIC in the specific treatment strategy of TRT combined with chemoimmunotherapy for ES-SCLC is essential. Additionally, to reduce lymphocyte toxicity and improve survival in ES-SCLC patients, we should prioritize the EDIC to develop immunoprotective therapeutic strategies.

## 6 Discussion and outlook

**Figure 2:**
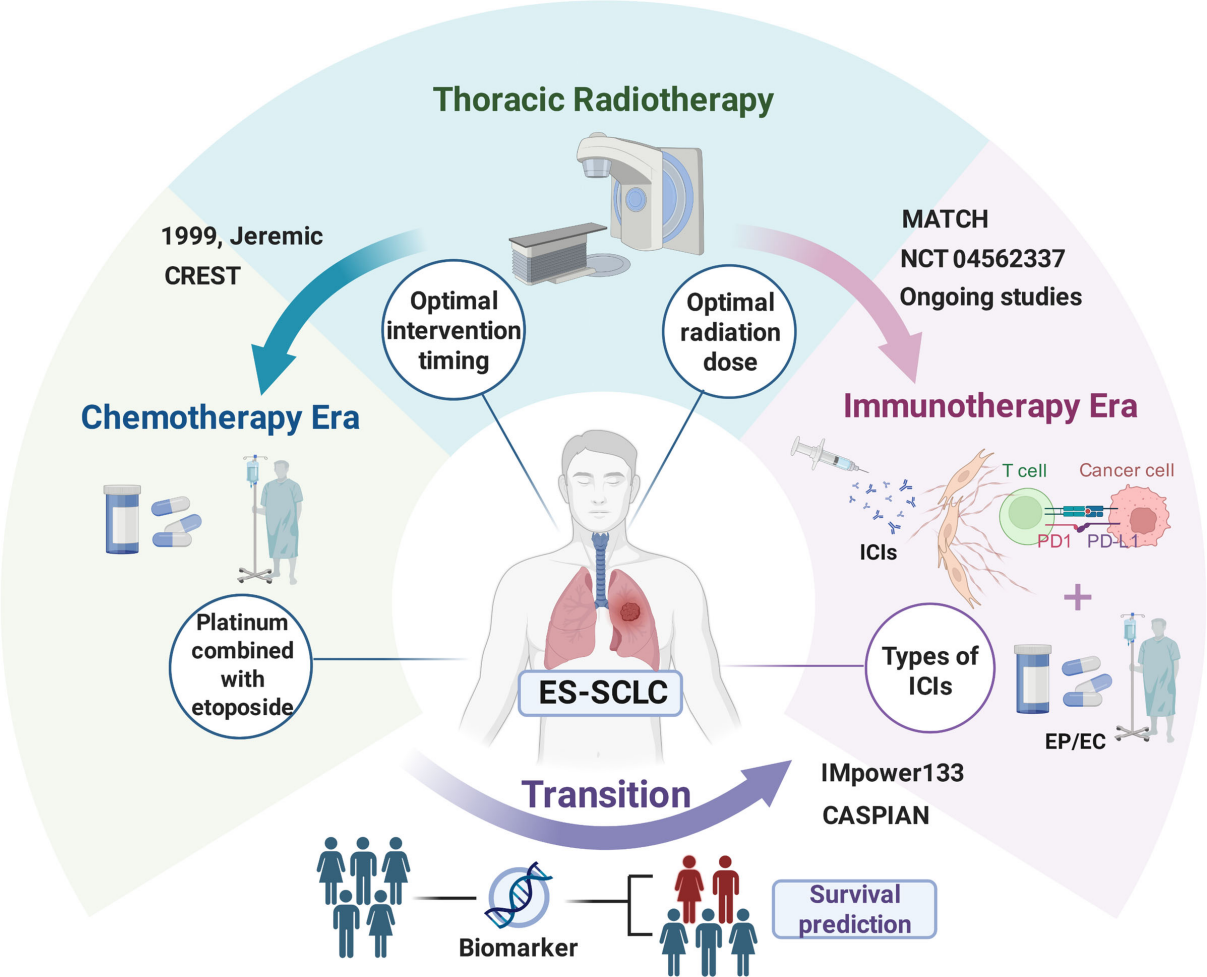
Treatment advances for extensive-stage small cell lung cancer. Created in BioRender. Li, Z. (2026) https://BioRender.com/sus2ecf.

Numerous clinical studies have confirmed that the addition of thoracic radiotherapy to the first-line chemoimmunotherapy for ES-SCLC patients seems to bring about additional survival benefits (**Figure 2**). However, most of these studies are single-center, retrospective, and have small sample. This results in insufficient clinical evidence supporting the conclusion, necessitating further prospective, multicenter clinical trials for validation.

At the same time, due to the significant variation in clinical characteristics and treatment courses among patients in retrospective studies, it has been challenging to explore the optimal dose, fractionation scheme, and timing of intervention for TRT. First, the optimal patient population benefiting from TRT remains poorly defined. Current evidence suggests that not all patients with ES-SCLC derive survival benefit from TRT. In clinical practice, those with residual intrathoracic disease after initial systemic therapy, low extrathoracic metastatic burden, and good performance status are more likely to benefit from consolidative TRT (88, 89). Conversely, patients with rapidly progressive systemic dissemination or poor baseline pulmonary function may experience limited survival gains. Therefore, baseline intrathoracic tumor burden, number of metastatic organs, response to induction chemoimmunotherapy, and dynamic biomarkers such as ctDNA warrant systematic evaluation in prospective studies. Second, the optimal timing of TRT remains unclear. Available evidence supports that TRT should be delivered before progressive disease occurs (90). However, whether it should be administered concurrently with or sequentially to first-line chemoimmunotherapy remains inconclusive. Most retrospective studies have adopted sequential TRT following 4–6 cycles of chemoimmunotherapy, whereas several prospective studies have explored concurrent TRT during the induction or early maintenance phase (46, 47). Third, the optimal dose and fractionation regimen of TRT remain undefined. Several retrospective studies have reported no significant survival differences between total doses (<30 Gy vs. >30 Gy), biologically effective doses (BED <60 Gy vs. >60 Gy), or conventional fractionation versus other fractionation schedules (43, 44). However, due to inherent selection bias, insufficient statistical power, and heterogeneity in target volume across these retrospective studies, true dose equivalence may not have been achieved. Notably, several completed phase II/III prospective studies have investigated different radiotherapy regimens and have preliminarily demonstrated that both sequential conventional or moderately hypofractionated thoracic radiotherapy after first-line chemoimmunotherapy, as well as concurrent hypofractionated or low-dose radiotherapy exhibit satisfactory efficacy with an acceptable safety profile (45–47). Furthermore, the results of several ongoing clinical trials also warrant attention (91). Fourth, target volume definition represents an important yet substantially understudied source of heterogeneity in existing studies (92). Therefore, future research should prospectively standardize target delineation principles to further clarify whether TRT should be directed only at residual intrathoracic lesions after induction therapy or should encompass the entire pretreatment intrathoracic disease extent.

Additionally, safety remains a critical concern warranting significant attention. Most studies have demonstrated that TRT combined with first-line chemoimmunotherapy exhibited acceptable toxicity. However, marked discrepancies in pneumonitis risk have been reported. These discrepancies may be attributable to heterogeneity in patient populations, random fluctuations due to small sample sizes, variations in dosimetric parameters, differences in the types of ICIs, and the lack of uniform diagnostic criteria for pneumonitis. Baseline patient characteristics, including pulmonary function, pre-existing interstitial lung changes, and smoking history, are well-established risk factors for pneumonitis. Furthermore, dosimetric parameters such as mean lung dose (MLD) and the volume of lung receiving ≥20 Gy (V20) are recognized predictors of radiation-induced pneumonitis (93). The lack of uniform eligibility criteria in retrospective studies has led to substantial heterogeneity in these factors that influence pneumonitis incidence. Interestingly, some studies suggest that PD-1 inhibitors, compared with PD-L1 inhibitors, may be associated with a relatively higher incidence of immune-related adverse events (94), which could increase the risk of pneumonitis attributable to the addition of TRT. Taken together, the risk of pneumonitis following TRT combined with chemoimmunotherapy is not a fixed effect but is likely modulated by the interplay of multiple clinical factors. In clinical practice, increased vigilance for pneumonitis is warranted when TRT is administered concurrently with immunotherapy or at higher doses.

Importantly, with the continuous advancement of radiotherapy techniques and the application of emerging technologies such as genomics and immunotherapy in ES-SCLC, the exploration of predictive biomarkers can help us further achieve precision and personalized treatment for ES-SCLC patients. However, current exploration of prognostic biomarkers for ES-SCLC patients remains extremely limited. On the one hand, most studies have focused on biomarkers in blood rather than tumor tissue biopsies. However, changes in peripheral blood do not fully reflect the responses within the tumor microenvironment. Therefore, exploring the mechanisms by which radiotherapy activates and modulates the unique tumor immune-suppressive microenvironment of SCLC through preclinical studies may be more conducive to identifying biomarkers in clinical settings. Additionally, baseline levels of immune cells in peripheral blood are influenced by multiple factors, such as age and gender. Therefore, using pre-treatment peripheral blood immune cell levels to predict patient survival appears inappropriate. Instead, attention should be focused on the dynamic changes in immune cell levels.

In summary, the novel treatment strategy combining first-line chemoimmunotherapy with TRT offers new hope for ES-SCLC patients. And the ongoing exploration of biomarkers predictive of survival provides a direction for precision medicine in ES-SCLC treatment.
